# Emergence of *Bordetella holmesii*–Associated Pertussis-Like Illness, Northern India, 2019–2023

**DOI:** 10.3201/eid3110.241659

**Published:** 2025-10

**Authors:** Nishant Shekhar, Deepa Sharma, Surajit Chakraborty, Rajesh Kumar, Rajneesh Singh Rawat, Nirmal Kaundal, Pawan Kumar, Tigran Avagyan, Deeksha Chauhan, Neha Jain, Megha Sharma, Arun Kumar, Vikas Gautam

**Affiliations:** Post Graduate Institute of Medical Education and Research, Chandigarh, India (N. Shekhar, R. Kumar, R.S. Rawat, D. Chauhan, V. Gautam); World Health Organization Country Office India, New Delhi, India (D. Sharma, N. Kaundal, T. Avagyan); University of Miami Miller School of Medicine, Miami, Florida, USA (S. Chakraborty); India Ministry of Health and Family Welfare, New Delhi (P. Kumar); Yatharth Hospital, Noida, India (N. Jain); All India Institute of Medical Science−Bilaspur, Bilaspur, India (M. Sharma)

**Keywords:** *Bordetella holmesii*, *Bordetella pertussis*, bacteria, respiratory infections, pertussis, real-time PCR, infections, epidemiology, India

## Abstract

We investigated *Bordetella holmesii* and *Bordetella pertussis* in 935 suspected pertussis cases in northern India (2019–2023) using PCR and serology. *B. holmesii* showed increased prevalence in pertussis cases, particularly in older children, highlighting its emerging role and the need for ongoing surveillance and adjusted prevention strategies.

Pertussis, caused by *Bordetella pertussis*, is a serious, vaccine-preventable respiratory illness (*1*,*2*). India’s immunization program recommends multiple tetanus-diphtheria-pertussis vaccine doses (*3*). The COVID-19 pandemic impacted vaccination rates and pertussis reporting across India, leading to a resurgence in pertussis cases (Appendix Figure 1). Concerns exist regarding other *Bordetella* species, such as *Bordetella holmesii*, a bacterium known to cause pertussis-like symptoms but not covered by current vaccines (*4*–*7*). Laboratory confirmation is crucial in differentiating *B. pertussis* from *B. holmesii* for accurate surveillance (*8*). Our study aimed to determine the incidence of *B. pertussis* and *B. holmesii* in pertussis-like cases in northern India.

## The Study

We analyzed 935 respiratory specimens collected from case patients clinically suspected of having pertussis in northern India during January 2019−August 2023. We sourced specimens from 2 cohorts: group I (n = 213), comprising patients hospitalized with acute respiratory illness at Postgraduate Institute of Medical Education and Research’s Advanced Pediatric Center; and group II (n = 722), obtained through the Vaccine-Preventable Disease Surveillance Network according to guidelines (*9*). We subjected 778 samples (178 from group I, 600 from group II) to real-time quantitative PCR (qPCR) (*10*) and examined 733 samples (101 from group I, 632 from group II) through serologic testing for pertussis toxin (PT) IgG using ELISA.

Of 778 swab samples tested by qPCR, we determined 383 (49.2%) to be positive for *Bordetella* IS*481*. Among those, we identified *B. pertussis* monoinfection in 52 (13.6%) samples, *B. holmesii* monoinfection in 143 (37.3%), and co-infection with both species in 15 (3.9%) samples (Table 1). Of note, *B. holmesii* positivity surpassed that of *B. pertussis* in 2022–2023 in the North India catchment. (Figure).

**Table 1:** Results of real-time quantitative PCR using the IS*481*, *ptx*S1, and hIS*1001* genes to differentiate *Bordetella* species in study of emergence of *Bordetella holmesii*–associated pertussis-like illness, northern India, 2019–2023

Category	No. samples
IS*481*	
Positive for *Bordetella* by multiplex PCR	197
Monoplex PCR, *ptx*S1	
Positive for *B. pertussis*	52
Negative	145
pIS*1001*	
Positive for *Bordetella* by multiplex PCR	17
Monoplex PCR, *ptx*S1	
Positive for *B. parapertussis*	2
Negative	15
IS*481* and hIS*1001*	
Positive for *Bordetella* by multiplex PCR	143
Monoplex PCR, *ptx*S1	
Positive for *B. pertussis* and *B. holmesii*	15
Negative for *B. holmesii*	128
IS*481* and pIS*1001*	
Multiplex PCR positive	26
Monoplex PCR, *ptx*S1	
Positive for *B. pertussis* and *B. parapertussis*	7
Negative	19
Total	778

**Figure:**
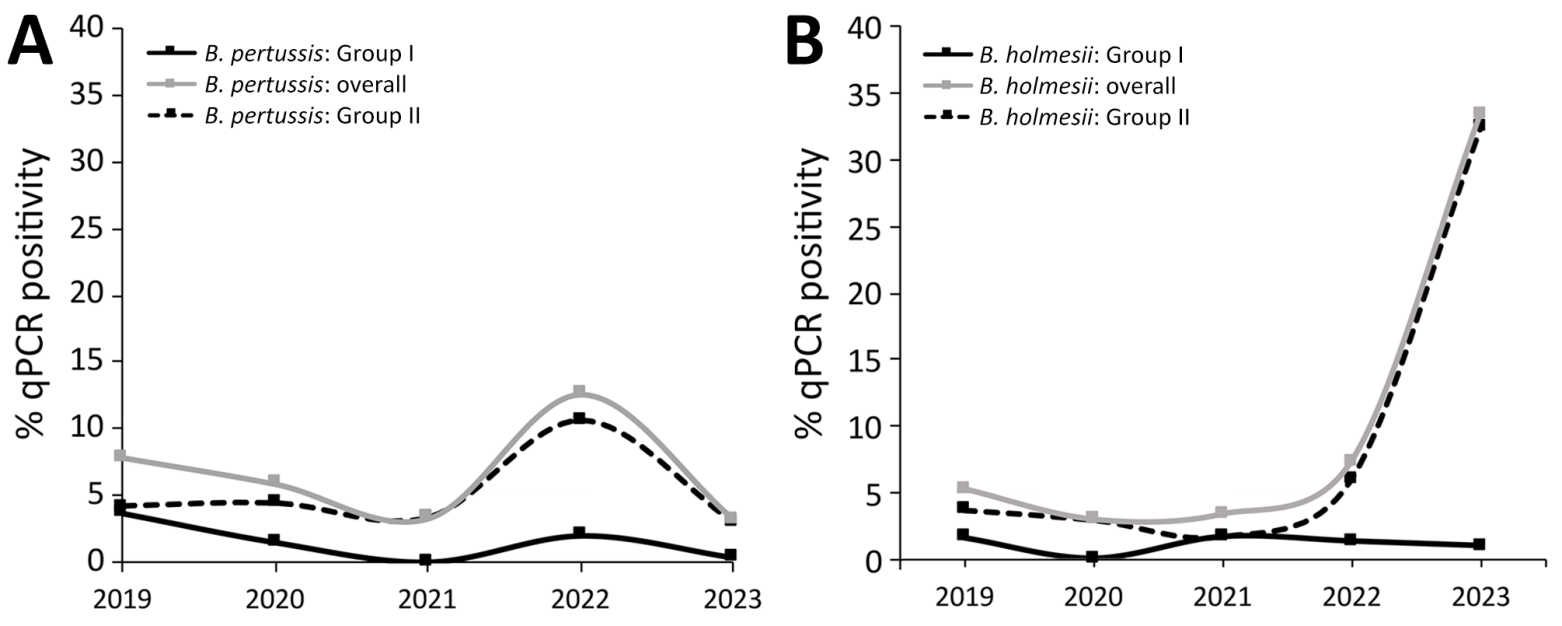
Yearwise trend of *Bordetella pertussis* and *B. holmesii* positivity from study of emergence of *B. holmesii*–associated pertussis-like illness, northern India, 2019–2023. Data based on real-time quantitative PCR using the IS*481*-*ptx*S1-hIS*1001* gene profile. Positiviity is shown overall and for 2 cohorts: group I (n = 213), patients hospitalized with acute respiratory illness at Postgraduate Institute of Medical Education and Research’s Advanced Pediatric Center; and group II (n = 722), obtained through the Vaccine-Preventable Disease Surveillance Network according to guidelines (*9*).

Both species predominantly affected infants <1 year of age. However, *B. holmesii* was significantly more prevalent in the 5–10-year age group (≈30% compared with ≈9% *B. pertussis*; χ^2^ = 16.22; p = 0.00102). The mean patient age for *B. holmesii* infection was 3.4 years and for *B. pertussis* was 1.9 years (Appendix Figure 2). We detected PT IgG with overall seroprevalence (positive + intermediate) in 232 (31.6%) of the 733 samples tested. We collected serology samples from 174 of the 210 cases that tested positive by qPCR.

Stratifying the qPCR and serology results of the samples for which qPCR and serology results were obtained, we identified 121 *B. holmesii* qPCR-confirmed cases, 43 of which we determined to be positive for PT IgG by ELISA. By that same method, we identified 41 qPCR-confirmed *B. pertussis* cases, determining 9 to be positive for PT IgG by ELISA (Table 2). Among 91 cases with known diphtheria-pertussis-tetanus vaccination status, 28 ELISA‐negative, unvaccinated case-patients (i.e., received no pertussis-containing vaccine [zero‐dose]) tested positive for *B. holmesii* monoinfection and 8 ELISA-negative, unvaccinated case-patients tested positive for *B. pertussis* monoinfection.

**Table 2:** Pertussis toxin IgG seroprevalence of real-time quantitative PCR–confirmed *Bordetella pertussis* and *B. holmesii* cases in study of emergence of *B. holmesii*–associated pertussis-like illness, northern India, 2019–2023

Serology	*B. pertussis*	*B. holmesii*	Co-infection with *B. pertussis* and *B. holmesii*
Positive	9	43	5
Negative	32	78	7
Total	41	121	12

## Conclusions

Our study highlights the emergence of *B. holmesii* as a key contributor to pertussis-like illness in northern India during 2021−2023. Species-specific qPCR revealed that *B. holmesii* detection rates overtook those of *B. pertussis* in the 2022–2023 period, particularly among children 5–10 years of age, suggesting an evolving epidemiology distinct from classic pertussis and echoing observations noted by researchers investigating other *B. holmesii* outbreaks (*11*–*13*). Our study demonstrates evidence of notable *B. holmesii* circulation in the India subcontinent, underscoring this pathogen’s potential role in co-infection and also as a primary etiologic agent, as demonstrated by the presence of this bacterium in unvaccinated PT IgG–negative case-patients (despite limitations in detailed clinical characterization of pertussis-like symptoms [e.g., cough duration, paroxysms] observed uniformly across cases).

In serology testing, PT IgG positivity (Table 1) among cases with *B. holmesii* monoinfection (qPCR-positive cases) might reflect recent *B. pertussis* exposure or vaccination. The absence of PT, pertactin, and fimbrial antigens from the *B. holmesii* genome and the lack of cross-protective immunity in whole-cell or acellular pertussis vaccines in animal models further reveals critical gaps in current immunization strategies (*4*,*5*). Although *B. holmesii* shares a filamentous hemagglutinin homologue and a conserved 66-kb pathogenicity island with *B. pertussis*, those shared elements have not yielded effective cross-protection or reliable serologic markers (*14*).

Our findings highlight the practicality of integrating molecular assays into routine surveillance to accurately distinguish *Bordetella* species and guide public health responses. Future research efforts should include more detailed clinical characterization of patients, population-based seroepidemiologic studies, and retrospective analysis of archived samples to clarify the historical prevalence of *B. holmesii*. Revisiting vaccine antigen composition to address nonpertussis *Bordetella* species might help close critical prevention gaps in pertussis-like disease control.

Appendix More information for emergence of *Bordetella holmesii*–associated pertussis-like illness, northern India, 2019–2023
