# Supplementary material for: Emergence of Bordetella holmesii–Associated Pertussis-Like Illness, Northern India, 2019–2023
Source: Emerg Infect Dis. 2025 Oct;31(10):2022–4. doi: 10.3201/eid3110.241659 (PMC12483014; doi:10.3201/eid3110.241659)
Supplement: Appendix — More information for emergence of Bordetella holmesii–associated pertussis-like illness, northern India, 2019–2023 [file 24-1659-Techapp-s1.pdf]

Article DOI: http://doi.org/10.3201/eid3110.241659

EID cannot ensure accessibility for supplementary materials supplied by authors. Readers who have difficulty accessing supplementary content should contact the authors for assistance.

Emergence of *Bordetella holmesii*–Associated Pertussis-Like Illness, Northern India, 2019–2023

Appendix

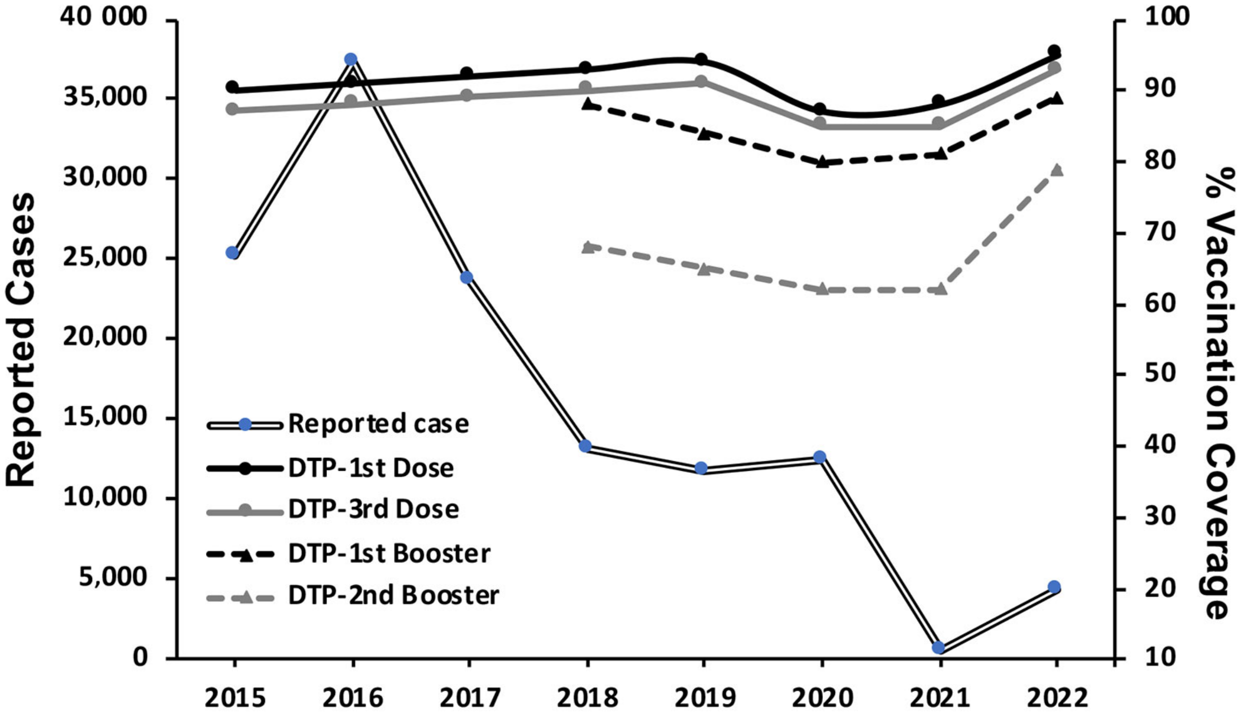

Appendix Figure 1. Year-wise trend of the number of reported cases of pertussis and DTP-antigen-containing vaccination coverage in India (accessed through https://immunizationdata.who.int), informing a study of the emergence of *Bordetella holmesii*–associated pertussis-like illness, northern India, 2019–2023.

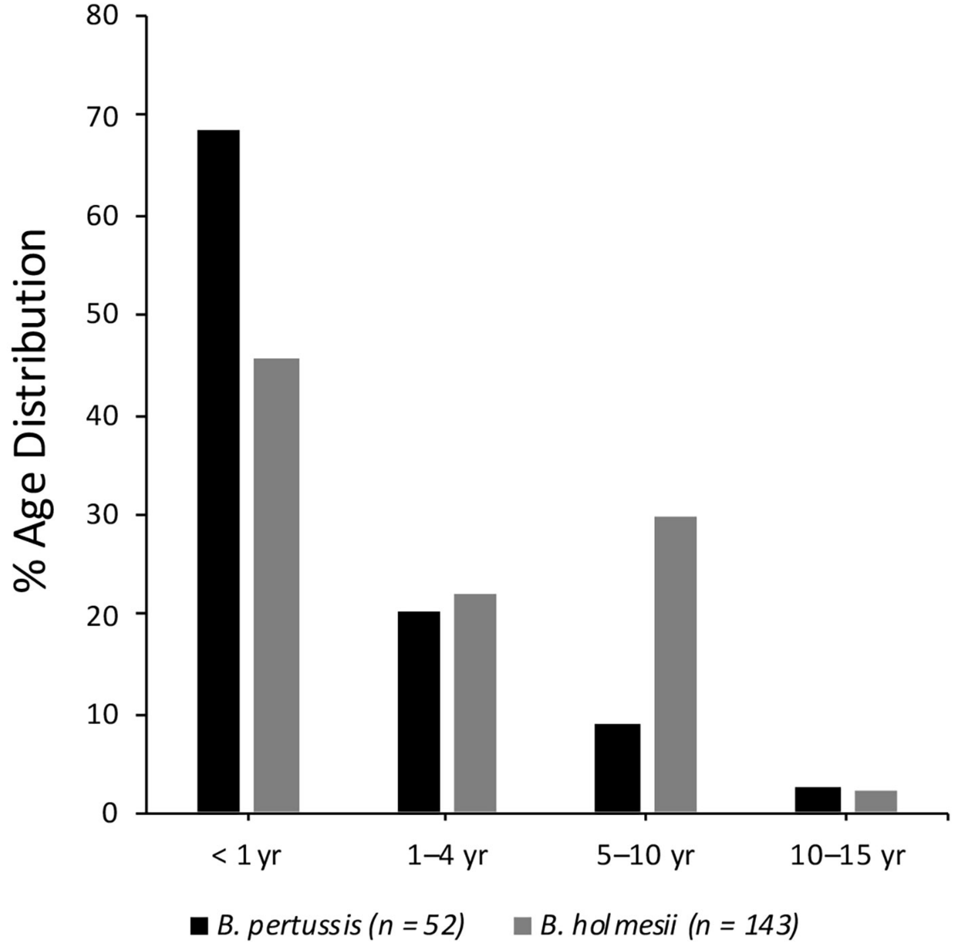

Appendix Figure 2. Percentage age distribution from a study of the emergence of *Bordetella holmesii*–associated pertussis-like illness, northern India, 2019–2023. Significant age group differences were observed between *Bordetella pertussis* and *B. holmesii* in the age groups <1 year and 5–10 years (χ^2 = 16.22; p = 0.00102).
